# Supplementary material for: MetaRibo-Seq measures translation in microbiomes
Source: Nat Commun. 2020 Jun 29;11:3268. doi: 10.1038/s41467-020-17081-z (PMC7324362; doi:10.1038/s41467-020-17081-z)
Supplement: Supplementary file 10 — Supplementary Data 7 [file 41467_2020_17081_MOESM10_ESM.zip › File2/Confidence_VeryHigh_Taxonomy/135980_out.krona.html]

Javascript must be enabled to view this page.

members
magnitude
magnitudeUnassigned
count
unassigned
taxon
rank

135980\_out

14

superkingdom
14
2

14
phylum
1239

186801
14
class

order
14
186802

9
family
186803

189330
genus
1

SRS062701\_contig\_number\_contig-100\_9930.9931

572511
8
genus

40520

SRS019068\_contig\_number\_67451SRS020869\_contig\_number\_contig-100\_3401.231978SRS024388\_contig\_number\_4238SRS078176\_contig\_number\_contig-100\_13093.92625SRS104311\_contig\_number\_contig-100\_269.266246SRS143342\_contig\_number\_contig-100\_2893.141105SRS147346\_contig\_number\_contig-100\_1845.357657SRS148159\_contig\_number\_contig-100\_1524.332298
8
species

541000
family
5

5

SRS076976\_contig\_number\_2803
genus
1263
1

species
1

SRS149181\_contig\_number\_22472
165186

657323

SRS015431\_contig\_number\_61765SRS057478\_contig\_number\_contig-100\_4410.27156SRS150029\_contig\_number\_14058
3
species
